# Supplementary material for: Polymer Nanocomposite Ultrafiltration Membranes: The Influence of Polymeric Additive, Dispersion Quality and Particle Modification on the Integration of Zinc Oxide Nanoparticles into Polyvinylidene Difluoride Membranes
Source: Membranes (Basel). 2020 Aug 24;10(9):197. doi: 10.3390/membranes10090197 (PMC7559267; doi:10.3390/membranes10090197)
Supplement: Supplementary file 1 [file membranes-10-00197-s001.pdf]

Supporting Information: Polymer Nanocomposite Ultrafiltration Membranes: the Influence of Polymeric Additive, Dispersion Quality and Particle Modification on the Integration of Zinc Oxide Nanoparticles into Polyvinylidene Difluoride Membranes

Thorsten van den Berg [1,,2], and Mathias Ulbricht [1,2,*]

[1] Lehrstuhl für Technische Chemie II, Universität Duisburg-Essen, 45141 Essen, Germany; thorsten.vandenberg@gmail.com
[2] Center for Nanointegration Duisburg-Essen (CENIDE), 47057 Duisburg, Germany
* Correspondence: mathias.ulbricht@uni-essen.de

Received: 28 July 2020; Accepted: 17 August 2020; Published: date

Table S1. Overview of cited studies on nanocomposite ultrafiltration membranes.

Source*	NP Type	Solvent	Additive	Dispersion method	Non-solvent	Pure water Permeability	Rejection	Macro void Formation	Hydrophilicity	Tensile Strength	Elongation at Break
Oh et al. 2009[11]	TiO_2	NMP	none	n.a.	iPrOH/ water	↓	n.a.	–	↑	n.a.	n.a.
Yan et al. 2006[13]	Al_2O_3	DMAc	PVP	stirring	EtOH/ water	↑	–	–	↑	↑	↑
Yu et al. 2009[14]	TiO_2	NMP	PVP	stirring	water	↗↘	–	↓	↑	↑	↑
Yari et al. 2019 [15]	SiO_2	NMP	none	sonication NP in solvent	water	↗↘	↑	↗↘	↑	n.a.	n.a.
Sun et al. 2019[16]	SiO_2	DMAc	PVP	stirring	water	↗↘	↑	↓	↑	n.a.	n.a.
Dong et al. 2012[17]	$Mg(OH)_2$	DMAc	PEG	NP in solvent	water	↗↘	–	↑	↑/↓ *	n.a.	n.a.
Liu et al. 2011[18]	Al_2O_3	NMP	H_2SO_4	stirring	water	↗↘	n.a.	↓	↑	↓	↓
Li et al. 2015[19]	TiO_2	DMAc	PVP	sonication NP in solvent	DMAc/ water	↑	n.a.	n.a.	↑	n.a.	n.a.
Moghadam et al. 2014[20]	TiO_2	DMAc	PEG	sonication of whole dope	water	↑	↑	↑	↑	↑	↑
Saraswathi et al. 2018[21]	MnO_2	NMP	PEG	sonication of whole dope	NMP/ water	↑	↑	↑	↑	↑	n.a.
Cao et al. 2006[22]	TiO_2	DMF	none	n.a.	water	↑	–	–	–	n.a.	n.a.
Liang et al. 2012[23]	ZnO	NMP	PVP, Glycerol	sonication of whole dope	water	↗↘	n.a.	–	↑	↑	↑
Song et al. 2012[24]	TiO_2	DMAc	PEG	n.a.	water	↑	↓	n.a.	↑	n.a.	n.a.
Yuliwati et al. 2011[25]	TiO_2	DMAc	LiCl	stirring	water	↗↘	↑	↓	n.a.	n.a.	n.a.

Wei et al. 2011[26]	TiO_2	DMAc	PVP	stirring	EtOH/ water	↑	n.a.	n.a.	n.a.	↑	↓
Zhang et al. 2017[27]	TiO_2	DMAc/ DMSO	PEG	stirring	water	↓	↑	n.a.	↑	n.a.	n.a.
Yuliwati et al. 2011[28]	TiO_2	DMAc	LiCl	stirring	water	↓	↑	↓	n.a.	n.a.	n.a.

Legend			
↑	Increase with NP conc.	–	no influence
↓	Decrease with NP conc.	n.a.	information not available
↗↘	increases at low NP conc., decreases at higher NP conc.	*	depends on PEG conc.

* for references see main paper.

Table S2. Overview on materials used in this study.

Substance	Type / Quality	Supplier / Manufacturer
1.4-dioxane	p.a.	Merck
2-butanol	ReagentPlus®, ≥ 99%w	Sigma-Aldrich
acetone	p.a.	Fluka
acetonitrile	p.a.	Merck
chloroform	p.a.	Acros
dichloromethane	p.a.	Aldrich
dimethyl sulfoxide	p.a.	Merck
dimethylacetamide	>98%	Fluka
dimethylformamide	Puris	AppliChem
Ethanol	p.a.	VWR Chemicals
ethyl acetate	p.a.	Fluka
methanol	p.a.	VWR Chemicals
methyl ethyl ketone	≥99.5%w pure	Aldrich
n-hexane	95%	Sigma-Aldrich
N-methyl-2-pyrrolidone	>99.5 %w pure	Merck
polyethylene glycole	$M_{w,av}$ ~ 35 kDa	Fluka
polyethylene oxide	$M_{w,av}$ ~ 100 kDa	Aldrich
polyvinylidene difluoride	Solef 6010 powder	Solvay Specialty Polymers
polyvinylpyrrolidone	Luvitec K-30; ≥ 95%w pure	BASF
p-xylene	p.a.	Merck
sodium azide	>99%w pure	Sigma-Aldrich
sodium chloride	ACS, ISO, Reag. Ph. Eur.	Merck
Tetrahydrofuran	p.a.	Merck
zinc oxide nanoparticle	VP ZnO 20; ≥ 99.5%w pure	Evonik Industries AG

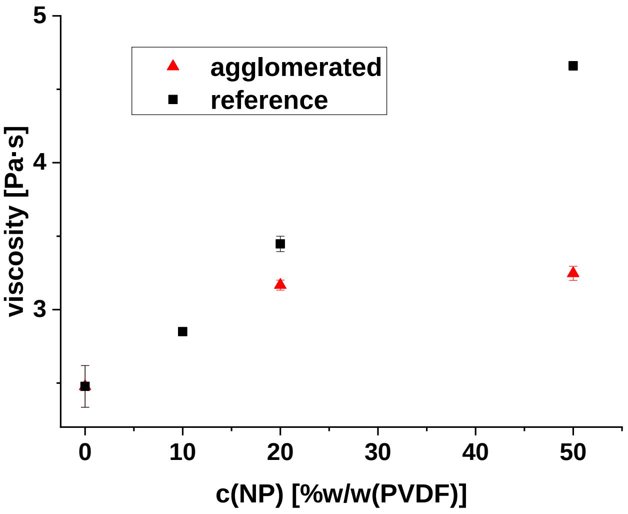

Figure S1. Effect of ZnO nanoparticle fraction onto viscosity of PVDF solutions in NMP obtained with (reference) and without sonication ("agglomerated"), measured at 15.9 s^{-1} and 20 °C.

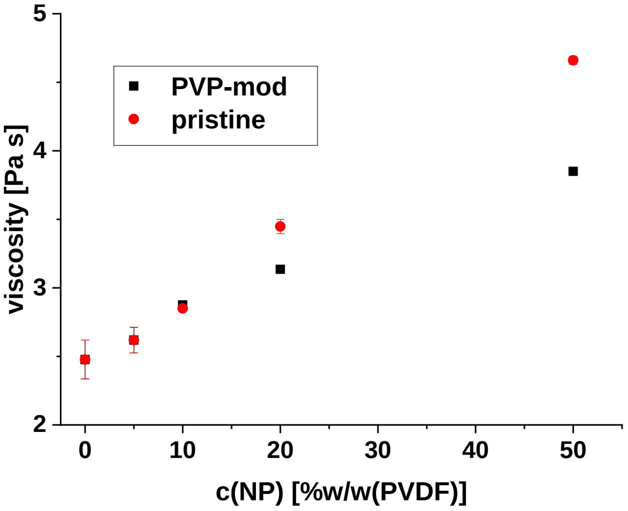

Figure S2. Viscosity of dope solutions (16 % PVDF in NMP) at 15.9 s^{-1}.

The ZnO particles with PVP modification lost 12.4 % of their mass during conditions simulating the dope solution preparation (cf. Figure 6 in main paper). Under the assumption that this mass consists solely of PVP the PVP concentration of the dope solutions can be calculated. The calculated PVP contents for the dope solutions shown in Figure S2 are presented in the following Table S2.

Table S3. Calculated PVP concentration in the dope solutions from Figure S2.

c(NP) [%/%(PVDF)]	$c(PVP)_{calculated}$ [%]
0	0
3	0.095
5	0.099
10	0.198
20	0.397
50	0.992

The viscosity increase caused by 1% PVP in the dope solution was calculated from the data (cf. Figure 1 in main paper) through linearization to be 0.44 ± 0.02 Pa s. The viscosity of the dope solution with ZnO-PVP particles was calculated by forming the product of the aforementioned viscosity increase for 1% PVP and the calculated PVP content of the dope solution (see Table S2) and then adding the viscosity of the dope solution without PVP for the adjusted ZnO content. The results are presented in Figure S3. It is apparent that the dope solutions with ZnO-PVP exhibit a lower viscosity than calculated values. Therefore, the difference in viscosity cannot be solely explained by the difference in ZnO and PVP content of the dope solutions.

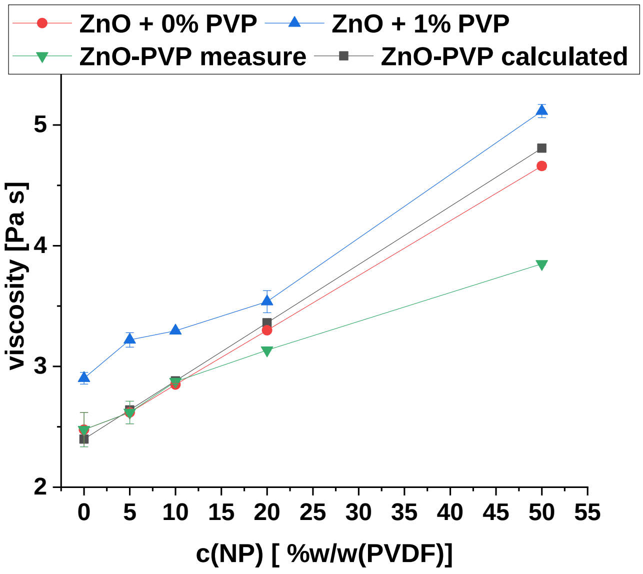

Figure S3. Comparison of the viscosity effect of PVP, pristine ZnO particles and ZnO-PVP particles with calculated viscosity increase for ZnO-PVP, measured at 15.9 s^{-1} and 20°C.

Table S4. Membrane performance data.

Membrane	Permeability [L/h m² bar]				Rejection [%]				n
	P_0	SD	P_1	SD	PEG 35kDa	SD	PEO 100kDa	SD	
1% PVP; no NP	1088	101	847	87	3	2	87	0	3
50% PVP-mod NP	957	77	759	81	29	15	80	1	2
1% PVP; 40% NP	536	41	411	25	49	4	81	1	2
1% PVP; 50% NP	500	232	400	222	57	21	78	11	33

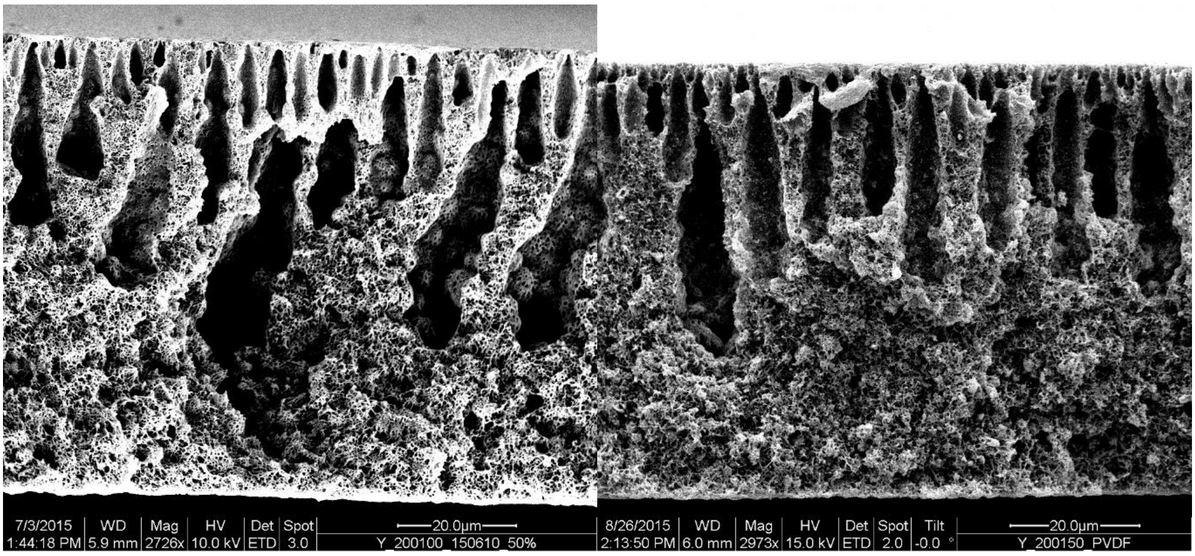

Figure S4. Cross-sections morphology of PVDF membranes obtained from dope solutions (20 % PVDF and 1 % PVP in NMP) precipitated in coagulation bath consisting of a 50 %Vol/50 %Vol mixture of NMP and water; left: no zinc oxide at 2726x magnification; right: 50 %w/w(PVDF) zinc oxide at 2973x magnification.
